# Non-steroidal anti-inflammatory agent use may not be associated with mortality of coronavirus disease 19

**DOI:** 10.1038/s41598-021-84539-5

**Published:** 2021-03-03

**Authors:** Jungchan Park, Seung-Hwa Lee, Seng Chan You, Jinseob Kim, Kwangmo Yang

**Affiliations:** 1 Department of Anesthesiology and Pain Medicine, Samsung Medical Center, Sungkyunkwan University School of Medicine, Seoul, South Korea; 2 Division of Cardiology, Department of Medicine, Heart Vascular Stroke Institute, Samsung Medical Center, Sungkyunkwan University School of Medicine, 81 Irwon-ro, Gangnam-gu, Seoul, 06351 South Korea; 3 Department of Biomedical Sciences, Ajou University Graduate School of Medicine, Suwon, South Korea; 4 Department of Epidemiology, School of Public Health, Seoul National University, Seoul, South Korea; 5 Center for Health Promotion, Samsung Medical Center, Sungkyunkwan University School of Medicine, 81 Irwon-ro, Gangnam-gu, Seoul, 06351 South Korea

**Keywords:** Microbiology, Health care, Medical research, Pathogenesis, Signs and symptoms

## Abstract

Non-steroidal anti-inflammatory drugs (NSAIDs) have been widely used in patients with respiratory infection, but their safety in coronavirus disease 19 (Covid-19) patients has not been fully investigated. We evaluated an association between NSAID use and outcomes of Covid-19. This study was a retrospective observational cohort study based on insurance benefit claims sent to the Health Insurance Review and Assessment Service of Korea by May 15, 2020. These claims comprised all Covid-19-tested cases and history of medical service use for the past 3 years in these patients. The primary outcome was all-cause mortality, and the secondary outcome was need for ventilator care. Among 7590 patients diagnosed with Covid-19, two distinct cohorts were generated based on NSAID or acetaminophen prescription within 2 weeks before Covid-19 diagnosis. A total of 398 patients was prescribed NSAIDs, and 2365 patients were prescribed acetaminophen. After propensity score matching, 397 pairs of data set were generated, and all-cause mortality of the NSAIDs group showed no significant difference compared with the acetaminophen group (4.0% vs. 3.0%; hazard ratio [HR], 1.33; 95% confidence interval [CI], 0.63–2.88; *P* = 0.46). The rate of ventilator care also did not show significantly different results between the two groups (2.0% vs. 1.3%; HR, 1.60; 95% CI 0.53–5.30; *P* = 0.42). Use of NSAIDs was not associated with mortality or ventilator care in Covid-19 patients. NSAIDs may be safely used to relieve symptoms in patients with suspicion of Covid-19.

## Introduction

In December 2019, a major outbreak of severe acute respiratory syndrome coronavirus 2 (SARS-Cov-2) in Wuhan City, China, was first reported. It was later characterized as coronavirus disease 19 (Covid-19) and has become a global threatening disease with more than 6,000,000 confirmed cases worldwide as of June 2020^1^. By then, more than 11,629 cases of Covid-19 had been diagnosed, and 273 deaths had been reported throughout Korea. Based on this, the government of Korea decided to share the world’s first de-identified Covid-19 nationwide patient data collected from the Korean National Health Insurance System for the purpose of investigation.

The primary site of infection in Covid-19 is the respiratory system. However, epidemiologic reports indicated that mortality of Covid-19 was much higher in patients with cardiovascular disease^2^, and the most serious complications of Covid-19 are those involving cardiovascular and respiratory systems, as well as sepsis^3,4^. Non-steroidal anti-inflammatory drugs (NSAIDs) have long been widely used for symptomatic relief of infected patients by controlling pain, fever, and inflammation, although safety concerns remain regarding harmful effects on the cardiovascular system^5^. Moreover, NSAID treatment was associated with pulmonary complication in patients with pneumonia^6^. The association between NSAID use and adverse outcome of Covid-19 has been previously evaluated, but limited data have been reported^7–9^. In this study, we used de-identified Covid-19 nationwide data from Korea to investigate the association between NSAID use to relieve symptoms before confirmed diagnosis of Covid-19 and mortality afterward. Our results may provide evidence for guidance on the use of NSAIDs in patients with symptoms suspicious of Covid-19.

## Results

**Figure 1:**
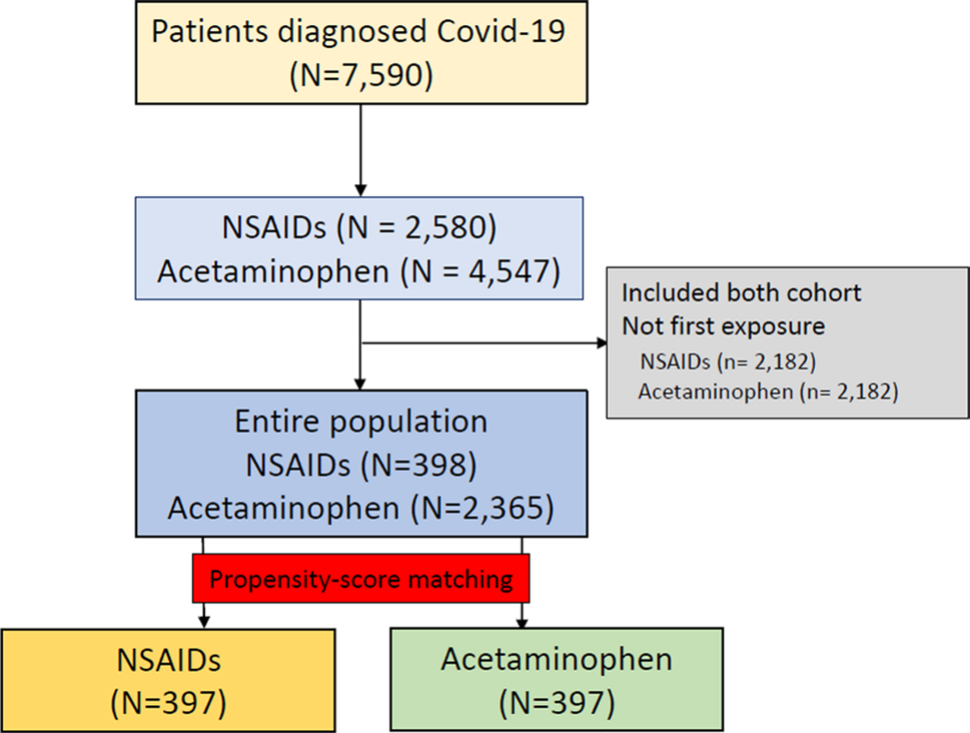
The flowchart of patients.

**Figure 2:**
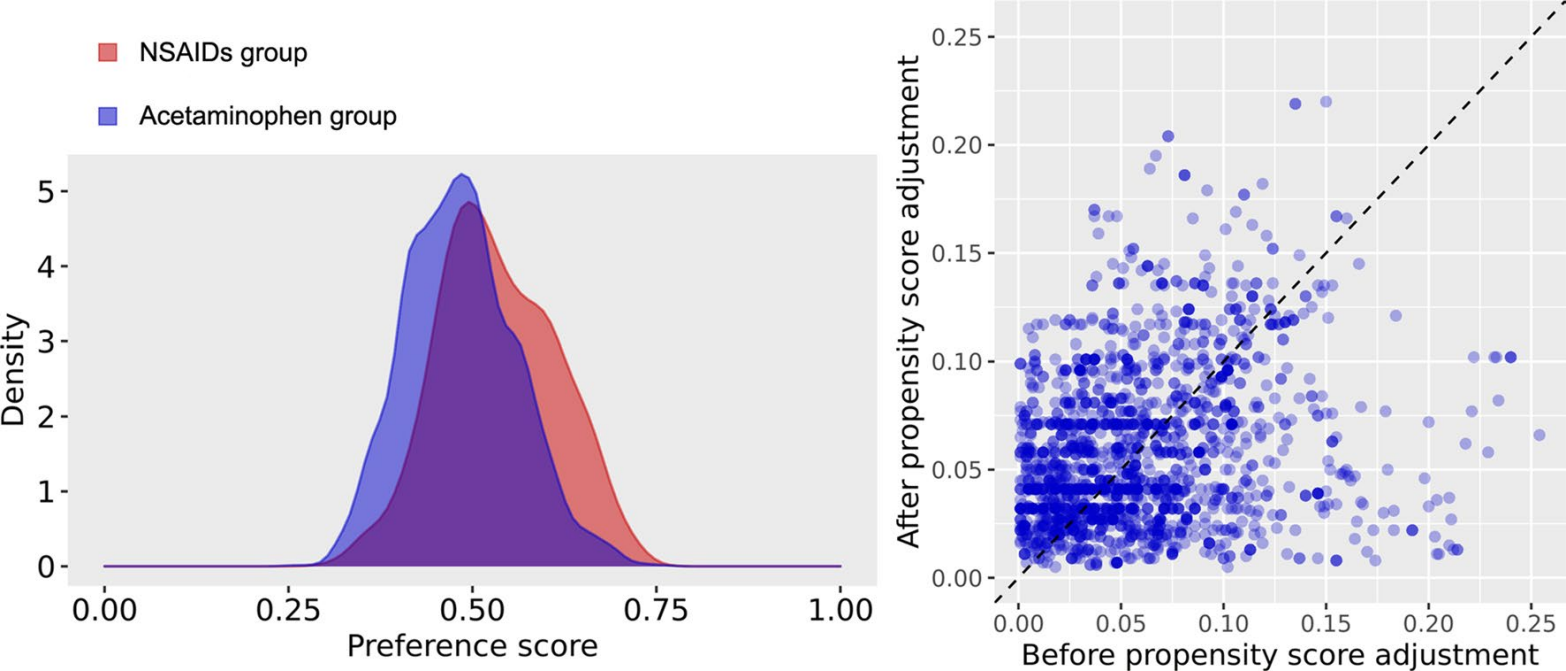
(**a**) Distributions of propensity score in each cohort before the propensity score matching. (**b**) Balance between variables before and after propensity score adjustment. Each dot represents the standardized difference of means for a single covariate before and after propensity score adjustment on the propensity score.

**Figure 3:**
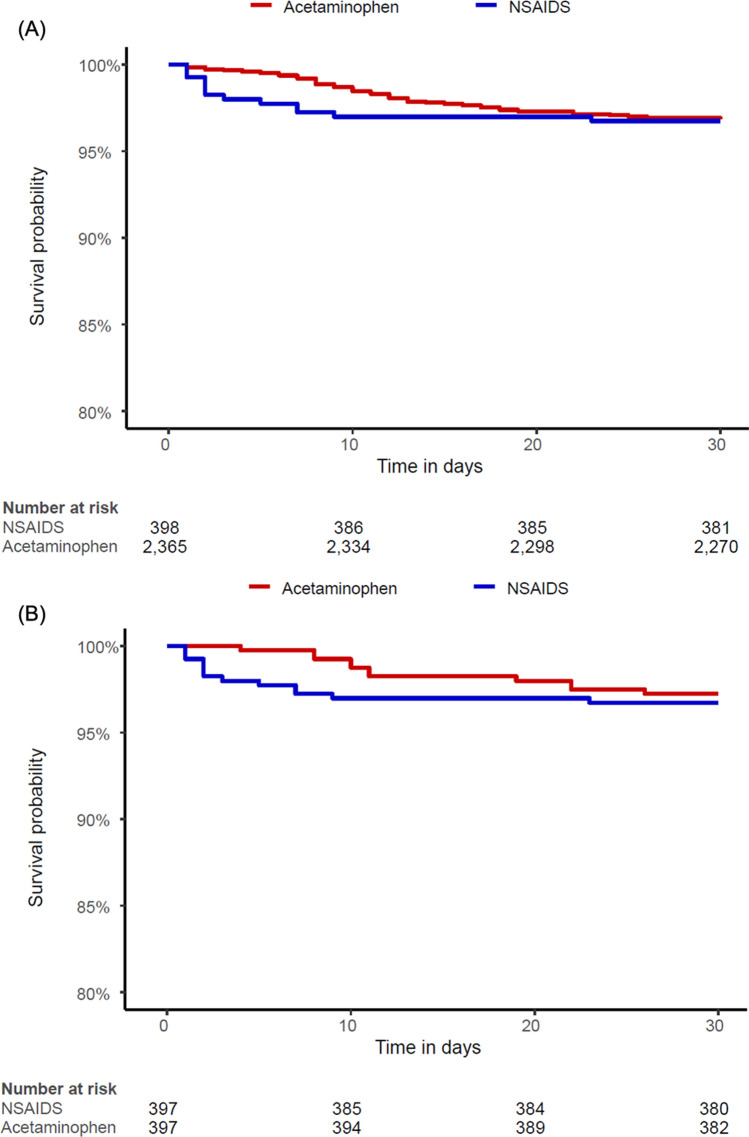
Kaplan–Meier curves for mortality in the (**a**) entire population and (**b**) propensity score matched population.

According to the data from the insurance benefit claims sent to HIRA until May 15, 2020, a total of 7590 patients was diagnosed with Covid-19. From these patients, 4069 patients with both of NSAIDs and acetaminophen prescriptions were excluded for cohorts generation. The target cohort consisted of 398 patients who were prescribed NSAIDs within 2 weeks before diagnosis, and the comparator cohort was generated by selecting 2365 patients who were prescribed acetaminophen in the same time frame (Fig. 1). The incidences of baseline characteristics are shown in Table 1. The median follow-up duration was 69 days (interquartile range 63–78) in the NSAID group and 71 days (interquartile range 58–80) in the acetaminophen group, and the maximum durations of follow-up were 113 days in the NSAID group and 111 days in the acetaminophen group. A total of 397 pairs of well-balanced groups was generated after propensity score matching (Table 1 and Fig. 2). In the propensity-score matched analysis, all-cause mortality of the NSAID group showed no significant difference from that of the acetaminophen group (4.0% vs. 3.0%; hazard ratio [HR], 1.33; 95% confidence interval [CI], 0.63–2.88; *P* = 0.69) (Table 2 and Fig. 3).

**Table 1:** Baseline characteristics**.**

	Before propensity score matching			After propensity score matching		
	NSAID (N = 398)	Acetaminophen (N = 2365)	SMD	NSAID (N = 397)	Acetaminophen (N = 397)	SMD
**Age group**						
20–24	14.3	12.8	0.04	14.4	11.6	0.08
25–29	11.1	12.1	− 0.03	11.1	13.6	− 0.08
30–34	3.8	6	− 0.1	3.8	5.3	− 0.07
35–39	4	6.2	− 0.1	4	6	− 0.09
40–44	5.7	6	− 0.01	5.6	5.8	0.01
45–49	9	8.2	0.03	9.1	10.6	− 0.05
50–54	9.5	10.9	− 0.04	9.6	8.1	0.05
55–59	10.6	9.2	0.04	10.6	9.1	0.05
60–64	8.3	8.8	− 0.02	8.3	9.3	− 0.04
65–69	5.5	6	− 0.02	5.3	6	− 0.03
70–74	3.3	4.6	− 0.07	3.3	5.3	− 0.1
75–79	6.3	3.6	0.12	6.3	3.5	0.13
80–84	3.3	2.7	0.04	3.3	2.8	0.03
85–89	3.8	2	0.11	3.8	2.5	0.07
90–94	1.5	0.9	0.05	1.5	0.5	0.1
Sex: female	58.3	60.8	− 0.05	58.2	63.2	− 0.1
**Medical history**						
Acute respiratory disease	76.6	69.4	0.16	76.6	77.3	− 0.02
Chronic liver disease	5.8	5.3	0.02	5.8	4.8	0.04
Chronic obstructive lung disease	2.5	1.9	0.04	2.5	0.8	0.14
Dementia	7.5	6.4	0.04	7.6	5.5	0.08
Depressive disorder	10.6	12	− 0.04	10.6	9.3	0.04
Diabetes mellitus	16.8	18.2	− 0.04	16.6	15.9	0.02
Gastroesophageal reflux disease	32.4	30.8	0.03	32.5	35.3	− 0.06
Gastrointestinal hemorrhage	2	2.3	− 0.02	2	1.3	0.06
Hyperlipidemia	31.4	32.5	− 0.02	31.2	29.5	0.04
Hypertensive disorder	28.6	24.3	0.1	28.7	27.7	0.02
Lesion of liver	3.3	2.2	0.07	3.3	1.8	0.1
Pneumonia	27.6	34.4	− 0.15	27.7	24.4	0.07
Psoriasis	1.8	0.9	0.08	1.8	1.3	0.04
Renal impairment	2.3	2.6	− 0.02	2.3	3	− 0.05
Rheumatoid arthritis	3.5	2.4	0.07	3.5	2.5	0.06
Schizophrenia	1.8	3.4	− 0.1	1.8	1.5	0.02
Urinary tract infectious disease	5.8	5.3	0.02	5.8	4.8	0.04
Viral hepatitis C	0.3	0.5	− 0.04	0.3	0.8	− 0.07
Visual system disorder	41.2	36.1	0.1	41.1	35.5	0.11
**Medical history: cardiovascular disease**						
Atrial fibrillation	1.3	0.9	0.04	1.3	0.8	0.05
Cerebrovascular disease	4.8	3	0.09	4.8	3.3	0.08
Heart disease	12.3	16.7	− 0.13	12.3	13.1	− 0.02
Heart failure	6	6.1	0	6	4.5	0.07
Ischemic heart disease						
Peripheral vascular disease	9.3	7.9	0.05	9.1	8.3	0.03
Venous thrombosis	2	0.6	0.12	2	1	0.08
**Medical history: neoplasms**						
Malignant neoplastic disease	4.5	4.9	− 0.02	4.5	4	0.03
Malignant tumor of breast	0.5	0.7	− 0.03	0.5	0.3	0.04
Malignant tumor of colon	0.3	0.5	− 0.04	0.3	0.5	− 0.04
Malignant tumor of lung	0.5	0.2	0.06	0.5	0.3	0.04

Data are presented as %.

*RAAS* renin–angiotensin–aldosterone system, *SMD* standardized mean difference.

**Table 2:** Clinical outcomes.

	Before propensity-score stratification				After propensity-score stratification			
	NSAID	Acetaminophen	Unadjusted	*P*-value	NSAID	Acetaminophen	Adjusted	*P*-value
	(N = 398)	(N = 2365)	HR (95% CI)		(N = 397)	(N = 397)	HR (95% CI)	
All-cause mortality	16 (4.0)	80 (3.4)	1.20 (0.68–1.99)	0.51	16 (4.0)	12 (3.0)	1.33 (0.63–2.88)	0.46

	NSAID	Acetaminophen	Unadjusted	*P*-value	NSAID	Acetaminophen	Adjusted	*P*-value
	(N = 396)	(N = 2355)	HR (95% CI)		(N = 395)	(N = 395)	HR (95% CI)	
Ventilator care	8 (2.0)	29 (1.2)	1.65 (0.70–3.44)	0.22	8 (2.0)	5 (1.3)	1.60 (0.53–5.30)	0.42

Data are presented as %.

*RAAS* renin–angiotensin–aldosterone system, *HR* hazard ratio, *CI* confidence interval.

For ventilator care comparison, 12 patients that needed ventilator care before Covid-19 diagnosis were excluded (Supplementary Fig. 1). The target cohort consisted of 396 patients on NSAID treatment, and the comparator cohort consisted of 2355 patients with acetaminophen treatment (Supplementary Table 1). After propensity score matching, a total of 395 pairs was generated, and we found no significant imbalance between the groups (Supplementary Table 1 and Supplementary Fig. 2). The incidence of ventilator care also showed no significant difference (2.0% vs. 1.3%; HR, 1.60; 95% CI, 0.53–5.30; *P* = 0.42) (Table 2 and Supplementary Fig. 3). In the sensitivity analysis, the use of NSAID was not associated with mortality and ventilator care during 30-day follow-up (Supplementary Table 2).

## Discussion

The present study showed that NSAID treatment in Covid-19 patients before diagnosis was not associated with increased mortality compared with acetaminophen treatment. The results of our study are in agreement with recently reported results and current recommendations^7,8,10^, suggesting that NSAIDs may be safely used in patients with symptoms suspicious for Covid-19.

NSAIDs are used globally to relieve pain and fever despite remaining concerns on cardiovascular safety, renal impairment, and upper gastrointestinal bleeding. Owing to these adverse effects, NSAIDs have been recommended to be avoided in patients with treatment-resistant hypertension, high risk for cardiovascular disease, and severe chronic kidney disease^11^. In addition, NSAID use in respiratory infection appeared to be associated with increased myocardial infarction and pleuropulmonary complications^5,6,12,13^. In this regard, use of acetaminophen, instead of NSAIDs, has been recommended by experts for symptomatic relief of Covid-19 patients.

In Covid-19 patients, cardiac comorbidities have consistently been reported to be a strong factor of disease severity. Reportedly, myocardial injury associated with SARS-CoV-2 occurred in 5 of the first 41 COVID-19 patients in Wuhan^14^, suggesting that cardiovascular protection should be considered during treatment of Covid-19^15^. Moreover, NSAID treatment in community-acquired pneumonia was shown to be associated with increased risk of pleuropulmonary complications^6^. However, there are limited data on the safety of NSAID treatment in Covid-19. A recent meta-analysis revealed only indirect evidence, and there is no direct evidence on severe adverse events, acute health care utilization, long-term survival, or quality of life as a result of use of NSAIDs in patients with Covid-19^7^. A small, single-center study reported that use of ibuprofen in Covid-19 patients led to no higher mortality or need for respiratory support compared with acetaminophen or no antipyretic agent^10^.

In the present study, we compared NSAID with acetaminophen use within 2 weeks before Covid-19 diagnosis and showed no significant difference in mortality or need for ventilator support. Our results suggest that NSAIDs could be a safe option for symptomatic relief even when Covid-19 is suspected, and further studies are needed to determine NSAID safety after diagnosis of Covid-19.

The results of this study should be interpreted considering the following limitations. First, this was a retrospective study, and despite our efforts to adjust all confounding factors by propensity score matching analysis, unmeasured factors might have affected the results. Second, the database used in this study retrieved information from insurance issued claims, so clinical presentation, symptoms, and hospital course could not be curated. Third, total amount of NSAIDs or acetaminophen was not considered in the analysis. In addition, a difference between types of NSAIDs or the use of NSAIDS without prescription were not considered. Lastly, the results of the current study are derived from a cohort of Koreans, and the results might be different in other countries. Despite these limitations, this study provides real-world data based evidence on the safety of NSAID use before diagnosis of Covid-19.

## Conclusion

Use of NSAIDs before Covid-19 diagnosis was not associated with increased mortality or need for ventilator care. Therefore, NSAIDs may safely be prescribed in patients with Covid-19.

## Methods

### Data curation

This is a retrospective observational cohort study conducted according to the principles of the Declaration of Helsinki and reported following the Strengthening the Reporting of Observational Studies in Epidemiology (STROBE) statement. The Institutional Review Board of Samsung Medical Center waived the need for approval and informed consent for this study (SMC 2020-04-135) since we used de-identified data of insurance benefit claims sent to the Health Insurance Review and Assessment Service of Korea (HIRA). This data set consists of all patients who were tested for Covid-19 using RT-PCR regardless of symptom in Korea until May 15, 2020. Our data set also includes the history of medical service used by these patients for the 3 years prior to the Covid-19 test. The data are shared as the Observational Medical Outcome Partnership Common Data Model (OMOP-CDM)^16,17^.

### Cohort definition and outcomes

All patients in study cohorts were diagnosed with Covid-19 using RT-PCR. The target cohort was generated by selecting patients who were prescribed NSAIDs within 2 weeks before Covid-19 diagnosis using RT-PCR. NSAID types was entered into the OHDSI repository (http://atlas-covid19.ohdsi.org/#/conceptset/454/conceptset-expression). For the comparator cohort, we selected patients with acetaminophen prescription within 2 weeks before Covid-19 diagnosis. Patients with both of NSAIDs and acetaminophen prescriptions were excluded from the analysis. The medical history of the cohorts were curated for the past 1 year, and the extracted incidence of baseline characteristics did not contain an exact number of patients to protect sensitive personal information and maintain de-identified data. The primary outcome was all-cause mortality. To compare the incidence of ventilator care as the secondary outcome, the cohorts were re-generated in the same manner but after excluding patients on ventilator care before Covid-19 diagnosis.

### Statistical analysis

OHDSI's open‐source software is publicly available on the GitHub repository (https://github.com/OHDSI/), and concept sets used to define baseline characteristics and study outcomes are also available (https://github.com/OHDSI/Covid-19/). The analysis was conducted under requests of investigators at https://hira-covid19.net. OHDSI analysis tools were built into the ATLAS interactive analysis platform and the OHDSI Methods Library R packages. ATLAS ver. 2.7.2 was used herein. As OHDSI CDM does not reveal exact numbers of patients for each covariate, we presented only incidences of baseline characteristics and used Cox regression analysis to compare outcomes. To minimize the effects of potential confounding factors and selection bias, we used large-scale propensity score matching with caliper 0.2 and generated a matched population to the cohorts without sample replacement. For the propensity score matching, we used Cyclops package in R programming to fit a large-scale regularized logistic regression^18^, and variables retained in the matching included age, female sex, and diagnosis codes with non-zero coefficients during 1 year prior to the diagnosis of Covid-19. The propensity score was stratified into 5 strata, and the Cox regression analysis retained strata. For sensitivity analysis, we conducted the same analysis for 30-day follow-up. Kaplan–Meier estimates were used to construct survival curves and compared using the log-rank test. All tests were two-tailed, and *P* < 0.05 was considered statistically significant.

## Supplementary Information

Supplementary Tables.

Supplementary Figure 1.

Supplementary Figure 2.

Supplementary Figure 3.

## References

[1] World Health Organization. Rolling updates on coronavirus disease (COVID-19) (2020). https://www.who.int/emergencies/diseases/novel-coronavirus-2019/events-as-they-happen. Accessed June 15, 2020.

[2] The Novel Coronavirus Pneumonia Emergency Response Epidemiology Team. Vital Surveillances: The Epidemiological Characteristics of an Outbreak of 2019 Novel Coronavirus Diseases (COVID-19)—China (2020). http://weekly.chinacdc.cn/en/article/id/e53946e2-c6c4-41e9-9a9b-fea8db1a8f51. Accessed June 15, 2020. PMC8392929 34594836

[3] Guan WJ (2020). Clinical characteristics of coronavirus disease 2019 in China. N. Engl. J. Med..

[4] Zhou F (2020). Clinical course and risk factors for mortality of adult inpatients with COVID-19 in Wuhan, China: A retrospective cohort study. Lancet.

[5] Coxib and Traditional NSAID Trialists' Collaboration (2013). Vascular and upper gastrointestinal effects of non-steroidal anti-inflammatory drugs: Meta-analyses of individual participant data from randomised trials. Lancet.

[6] Basille D (2018). Nonsteroidal antiinflammatory drug use and clinical outcomes of community-acquired pneumonia. Am. J. Respir. Crit. Care Med..

[7] Russell B, Moss C, Rigg A, Van Hemelrijck M (2020). COVID-19 and treatment with NSAIDs and corticosteroids: Should we be limiting their use in the clinical setting?. Ecancermedicalscience.

[8] Little P (2020). Non-steroidal anti-inflammatory drugs and covid-19. BMJ.

[9] Capuano A, Scavone C, Racagni G, Scaglione F, Italian Society of Pharmacology (2020). NSAIDs in patients with viral infections, including Covid-19: Victims or perpetrators?. Pharmacol. Res..

[10] Rinott E, Kozer E, Shapira Y, Bar-Haim A, Youngster I (2020). Ibuprofen use and clinical outcomes in COVID-19 patients. Clin. Microbiol. Infect..

[11] Szeto CC (2020). Non-steroidal anti-inflammatory drug (NSAID) therapy in patients with hypertension, cardiovascular, renal or gastrointestinal comorbidities: Joint APAGE/APLAR/APSDE/APSH/APSN/PoA recommendations. Gut.

[12] Wen YC (2017). Acute respiratory infection and use of nonsteroidal anti-inflammatory drugs on risk of acute myocardial infarction: A nationwide case-crossover study. J. Infect. Dis..

[13] Schjerning AM, McGettigan P, Gislason G (2020). Cardiovascular effects and safety of (non-aspirin) NSAIDs. Nat. Rev. Cardiol..

[14] Huang C (2020). Clinical features of patients infected with 2019 novel coronavirus in Wuhan, China. Lancet.

[15] Zheng YY, Ma YT, Zhang JY, Xie X (2020). COVID-19 and the cardiovascular system. Nat. Rev. Cardiol..

[16] You SC, Gundlapalli AV, Jaulent MC, Zhao D (2018). MEDINFO 2017: Precision Healthcare Through Informatics.

[17] Overhage JM, Ryan PB, Reich CG, Hartzema AG, Stang PE (2012). Validation of a common data model for active safety surveillance research. J. Am. Med. Inform. Assoc..

[18] Suchard MA, Simpson SE, Zorych I, Ryan P, Madigan D (2013). Massive parallelization of serial inference algorithms for a complex generalized linear model. ACM Trans. Model. Comput. Simul..

