# Supplementary material for: Non-steroidal anti-inflammatory agent use may not be associated with mortality of coronavirus disease 19
Source: Sci Rep. 2021 Mar 3;11:5087. doi: 10.1038/s41598-021-84539-5 (PMC7930278; doi:10.1038/s41598-021-84539-5)
Supplement: Supplementary file 1 — Supplementary Tables. [file 41598_2021_84539_MOESM1_ESM.docx]

**Non-Steroidal Anti-Inflammatory Agent Use May Not Be Associated with Mortality of Coronavirus Disease 19**

**Jungchan Park,^1^ Seung-Hwa Lee,^2^ Seng Chan You,^3^ Jinseob Kim,^4^ and Kwangmo Yang^5^**

^1^Department of Anesthesiology and Pain Medicine, Samsung Medical Center, Sungkyunkwan University School of Medicine, Seoul, Korea

^2^Division of Cardiology, Department of Medicine, Heart Vascular Stroke Institute, Samsung Medical Center, Sungkyunkwan University School of Medicine, Seoul, Korea

^3^Department of Biomedical Sciences, Ajou University Graduate School of Medicine, Suwon, Korea

^4^Department of Epidemiology, School of Public Health, Seoul National University, Seoul, Korea

^5^Center for Health Promotion, Samsung Medical Center, Sungkyunkwan University School of Medicine, Seoul, Korea

Drs. J. Park and SH Lee contributed equally to this work as co-first authors.

Drs. SH Lee and K. Yang contributed equally to this work as corresponding authors.

**Running title:** Renin-Angiotensin-Aldosterone System Inhibitors in Covid-19 Patients

**Funding:** This research was funded by the Ministry of Health and Welfare, Korea (grant number: HI19C0811).

**Corresponding author at:**

Seung-Hwa Lee, MD, Professor, Division of Cardiology, Department of Medicine, Heart Vascular Stroke Institute, Samsung Medical Center, Sungkyunkwan University School of Medicine, 81 Irwon-ro, Gangnam-gu, Seoul, 06351, Korea

Tel: +82-2-3410-3214; Fax: +82-2-3410-3897; E-mail: shuaaa.lee@samsung.com

&

Kwangmo Yang, MD, Professor, Center for Health Promotion, Samsung Medical Center, Sungkyunkwan University School of Medicine, 81 Irwon-ro, Gangnam-gu, Seoul, 06351, Korea

Tel: +82-2-3410- ; Fax: +82-2-3410- ; E-mail: kmhi.yang@samsung.com

**Supplemental Table 1. Baseline Characteristics of the Cohorts for ventilator care analysis**

|  | **Before propensity score matching** | | | **After propensity score matching** | | |
| --- | --- | --- | --- | --- | --- | --- |
|  | **NSAID** | **Acetaminophen** | **SMD** | **NSAID** | **Acetaminophen** | **SMD** |
|  | **(N = 396)** | **(N = 2,355)** |  | **(N = 395)** | **(N = 395)** |  |
| **Age group** |  |  |  |  |  |  |
| 20-24 | 14.3 | 12.8 | 0.04 | 14.4 | 12.2 | 0.07 |
| 25-29 | 11.1 | 12.1 | -0.03 | 11.1 | 13.7 | -0.08 |
| 30-34 | 3.8 | 6 | -0.1 | 3.8 | 5.3 | -0.07 |
| 35-39 | 4 | 6.2 | -0.1 | 4.1 | 6.1 | -0.09 |
| 40-44 | 5.8 | 5.9 | 0 | 5.8 | 5.6 | 0.01 |
| 45-49 | 9 | 8.2 | 0.03 | 9.1 | 10.4 | -0.04 |
| 50-54 | 9.5 | 10.9 | -0.04 | 9.6 | 8.1 | 0.05 |
| 55-59 | 10.6 | 9.2 | 0.04 | 10.6 | 9.1 | 0.05 |
| 60-64 | 8.3 | 8.8 | -0.02 | 8.1 | 9.1 | -0.04 |
| 65-69 | 5.5 | 6 | -0.02 | 5.3 | 6.1 | -0.03 |
| 70-74 | 3.3 | 4.6 | -0.07 | 3.3 | 5.3 | -0.1 |
| 75-79 | 6.3 | 3.6 | 0.12 | 6.1 | 3.5 | 0.12 |
| 80-84 | 3.3 | 2.7 | 0.04 | 3.3 | 2.8 | 0.03 |
| 85-89 | 3.8 | 2 | 0.11 | 3.8 | 2.3 | 0.09 |
| 90-94 | 1.5 | 0.9 | 0.05 | 1.5 | 0.5 | 0.1 |
| **Sex: Female** | 58.3 | 60.8 | -0.05 | 58.5 | 63 | -0.09 |
| **Medical history** |  |  |  |  |  |  |
| Acute respiratory disease | 76.6 | 69.4 | 0.16 | 76.7 | 77.5 | -0.02 |
| Chronic liver disease | 5.8 | 5.3 | 0.02 | 5.8 | 4.8 | 0.04 |
| Chronic obstructive lung disease | 2.5 | 1.9 | 0.04 | 2.5 | 0.8 | 0.14 |
| Dementia | 7.5 | 6.4 | 0.04 | 7.6 | 5.3 | 0.09 |
| Depressive disorder | 10.6 | 12 | -0.04 | 10.6 | 9.1 | 0.05 |
| Diabetes mellitus | 16.8 | 18.2 | -0.04 | 16.7 | 15.4 | 0.03 |
| Gastroesophageal reflux disease | 32.4 | 30.8 | 0.03 | 32.4 | 35.4 | -0.06 |
| Gastrointestinal hemorrhage | 2 | 2.3 | -0.02 | 2 | 1.3 | 0.06 |
| Hyperlipidemia | 31.4 | 32.5 | -0.02 | 31.1 | 29.1 | 0.04 |
| Hypertensive disorder | 28.6 | 24.3 | 0.1 | 28.4 | 27.1 | 0.03 |
| Lesion of liver | 3.3 | 2.2 | 0.07 | 3.3 | 1.8 | 0.1 |
| Pneumonia | 27.6 | 34.4 | -0.15 | 27.3 | 24.3 | 0.07 |
| Psoriasis | 1.8 | 0.9 | 0.08 | 1.8 | 1.3 | 0.04 |
| Renal impairment | 2.3 | 2.6 | -0.02 | 2.3 | 2.8 | -0.03 |
| Rheumatoid arthritis | 3.5 | 2.4 | 0.07 | 3.5 | 2.5 | 0.06 |
| Schizophrenia | 1.8 | 3.4 | -0.1 | 1.8 | 1.5 | 0.02 |
| Urinary tract infectious disease | 5.8 | 5.3 | 0.02 | 5.6 | 4.6 | 0.05 |
| Viral hepatitis C | 0.3 | 0.5 | -0.04 | 0.3 | 0.8 | -0.07 |
| Visual system disorder | 41.2 | 36.1 | 0.1 | 41 | 35.9 | 0.1 |
| **Medical history: Cardiovascular disease** |  |  |  |  |  |  |
| Atrial fibrillation | 1.3 | 0.9 | 0.04 | 1 | 0.8 | 0.03 |
| Cerebrovascular disease | 4.8 | 3 | 0.09 | 4.8 | 2.8 | 0.11 |
| Heart disease | 12.3 | 16.7 | -0.13 | 11.9 | 12.4 | -0.01 |
| Heart failure | 6 | 6.1 | 0 | 5.8 | 4.3 | 0.07 |
| Ischemic heart disease | 6 | 9.5 | -0.13 | 5.8 | 5.6 | 0.01 |
| Peripheral vascular disease | 9.3 | 7.9 | 0.05 | 9.1 | 8.1 | 0.04 |
| Venous thrombosis | 2 | 0.6 | 0.12 | 2 | 0.8 | 0.11 |
| **Medical history: Neoplasms** |  |  |  |  |  |  |
| Malignant neoplastic disease | 4.5 | 4.9 | -0.02 | 4.6 | 4.1 | 0.03 |
| Malignant tumor of breast | 0.5 | 0.7 | -0.03 | 0.5 | 0.3 | 0.04 |
| Malignant tumor of colon | 0.3 | 0.5 | -0.04 | 0.3 | 0.5 | -0.04 |
| Malignant tumor of lung | 0.5 | 0.2 | 0.06 | 0.5 | 0.3 | 0.04 |

Data are presented as %.

Abbreviations: RAAS, renin-angiotensin-aldosterone system; SMD, standardized mean difference

**Supplemental Table 2. Sensitivity analysis on the association between NSAID use and outcomes during 30-day follow-up**

|  | **HR (95% CI)** | **P-value** |
| --- | --- | --- |
| **All-cause mortality** | 1.13 (0.63-1.91) | 0.67 |
| **Ventilator care** | 1.39 (0.59-2.96) | 0.42 |

Abbreviations: HR, hazard ratio; CI, confidence interval
