# Supplementary material for: Non-steroidal anti-inflammatory agent use may not be associated with mortality of coronavirus disease 19
Source: Sci Rep. 2021 Mar 3;11:5087. doi: 10.1038/s41598-021-84539-5 (PMC7930278; doi:10.1038/s41598-021-84539-5)

**Non-Steroidal Anti-Inflammatory Agent Use May Not Be Associated with Mortality of Coronavirus Disease 19**

**Jungchan Park,^1^ Seung-Hwa Lee,^2^ Seng Chan You,^3^ Jinseob Kim,^4^ and Kwangmo Yang^5^**

^1^Department of Anesthesiology and Pain Medicine, Samsung Medical Center, Sungkyunkwan University School of Medicine, Seoul, Korea

^2^Division of Cardiology, Department of Medicine, Heart Vascular Stroke Institute, Samsung Medical Center, Sungkyunkwan University School of Medicine, Seoul, Korea

^3^Department of Biomedical Sciences, Ajou University Graduate School of Medicine, Suwon, Korea

^4^Department of Epidemiology, School of Public Health, Seoul National University, Seoul, Korea

^5^Center for Health Promotion, Samsung Medical Center, Sungkyunkwan University School of Medicine, Seoul, Korea

Drs. J. Park and SH Lee contributed equally to this work as co-first authors.

Drs. SH Lee and K. Yang contributed equally to this work as corresponding authors.

**Running title:** Renin-Angiotensin-Aldosterone System Inhibitors in Covid-19 Patients

**Funding:** This research was funded by the Ministry of Health and Welfare, Korea (grant number: HI19C0811).

**Corresponding author at:**

Seung-Hwa Lee, MD, Professor, Division of Cardiology, Department of Medicine, Heart Vascular Stroke Institute, Samsung Medical Center, Sungkyunkwan University School of Medicine, 81 Irwon-ro, Gangnam-gu, Seoul, 06351, Korea

Tel: +82-2-3410-3214; Fax: +82-2-3410-3897; E-mail: shuaaa.lee@samsung.com

&

Kwangmo Yang, MD, Professor, Center for Health Promotion, Samsung Medical Center, Sungkyunkwan University School of Medicine, 81 Irwon-ro, Gangnam-gu, Seoul, 06351, Korea

Tel: +82-2-3410- ; Fax: +82-2-3410- ; E-mail: kmhi.yang@samsung.com

**Supplementary Fig. 1.** The flowchart of patients for ventilator care analysis


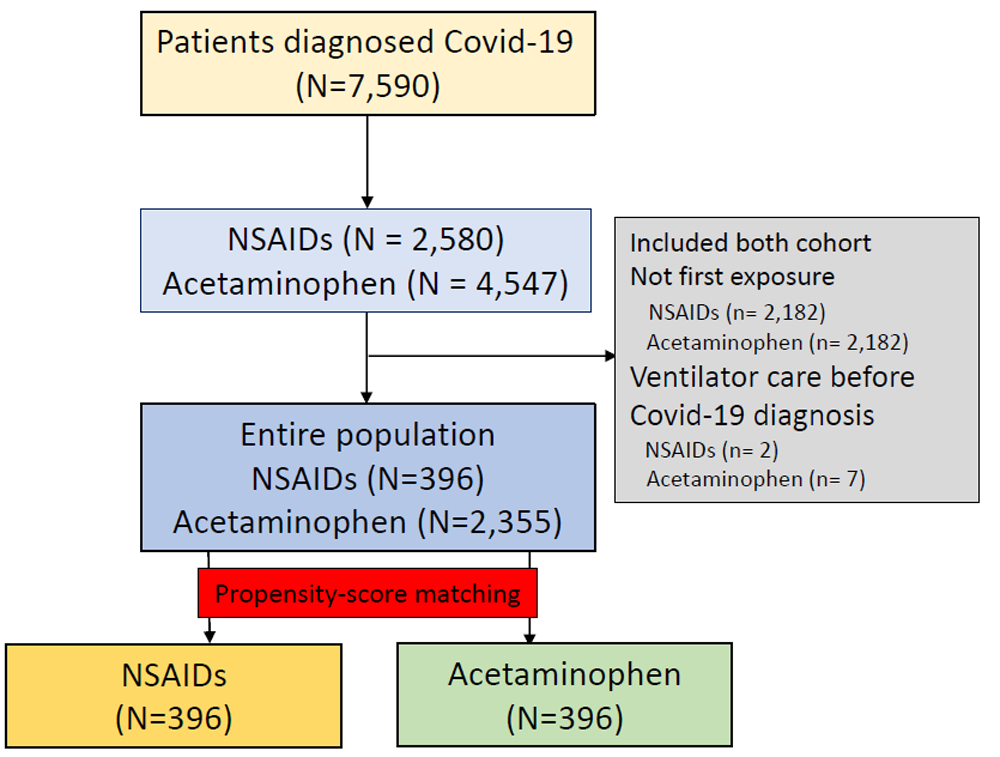

Supplement: Supplementary file 2 — Supplementary Figure 1. [file 41598_2021_84539_MOESM2_ESM.docx]
